# Application of ion-exchange dynamic conditions in the recovery of precious metals from refining waste

**DOI:** 10.1038/s41598-024-66086-x

**Published:** 2024-07-01

**Authors:** Karolina Goc, Grzegorz Benke, Joanna Kluczka, Karolina Pianowska, Joanna Malarz, Katarzyna Leszczyńska-Sejda

**Affiliations:** 1 Hydroelectrometallurgy Centre, Łukasiewicz Research Network-Institute of Non-Ferrous Metals, Sowińskiego 5, 44-100 Gliwice, Poland; 2 Department of Inorganic Chemistry, Analytical Chemistry and Electrochemistry, Faculty of Chemistry, Silesian University of Technology, B. Krzywoustego 6, 44-100 Gliwice, Poland

**Keywords:** Precious metals, Sorption, Elution, Ion-exchange resin, Critical raw materials, Hydrometallurgical recovery of metals, Chemical engineering, Chemical engineering, Inorganic chemistry

## Abstract

The objective of this study was to assess the potential for recovering precious metals from technological solutions using an ion-exchange dynamic method. Precious metals like platinum, palladium, rhodium, and gold are essential materials in various industries such as: automotive, electronics, pharmaceuticals, and jewellery. Due to their limited occurrence in primary sources, there is a growing trend in the market to extract these metals from secondary sources. The research involved conducting sorption and elution tests under different parameters to investigate their impact on the process in dynamic conditions. Additionally, an attempt was made to calculate the operational and total capacity of the resins, which has not been done previously for industrial solutions. The results showed that using Puromet MTS9200, Puromet MTS9850, and Lewatit MonoPlus MP600 resins, the sorption process could be effectively carried out in dynamic conditions with a contact time of 5 min between the technological solution and the resin bed. For optimal elution, the contact time between the eluent solution and the bed should range between 10 and 30 min. To improve rhodium sorption efficiency, it was found that neutralizing the technological solution to a pH of approximately 7 and using Lewatit MonoPlus MP600 resin could be beneficial.

## Introduction

Cars, jewellery, computers, electronics are an integral part of everyday life and share a few common elements—in this case in all of them precious metals are used as one of the most important parts^1–5^. Platinum, palladium, rhodium and gold are used in many materials due to their aesthetic, but also because they are characterized by exceptional resistance to corrosion by a wide range of liquid and gaseous substances and relative stability at high temperatures, in conditions in which other metals would quickly oxidize^6,7^. For users such properties are highly needed, unfortunately the utilization of the used products is a great challenge. Due to the primary sources being centralized in the specific areas on the globe, such as South Africa, Canada, Russia, the USA, Zimbabwe and China^8,9^, the rest of the world either have to import the precious metals and/or obtain them from different sources. This problem intertwines with the rising consumerism of the goods on the market, with new products being manufactured every year and the old one being thrown away^2,10–13^. Due to their low occurrence and high demand, precious metals already have been on the list of critical raw materials for the European Union for several years. Therefore, the world changed their perspective on obtaining the precious metals from primary source—which would save the deposits—to secondary sources—automotive catalyst, used jewellery, WEEE and others^14–16^.

Recovering specific elements is not easy, especially when said metals have incredibly good chemical and thermal resistance^17^. The waste containing precious metals are also a multicomponent material, which further on complicates the recycling process^18^. Nevertheless, there are several steps which are often repeated, no matter the type of waste—classification, dismantling, grinding, crushing, smelting, leaching and further raffination^1,19–23^. Precious metals like platinum, palladium, rhodium and gold can be only recovered using hydrometallurgical methods, therefore the leaching step is one of the most important part, which determines the use of future techniques. In terms of such difficult metals, several leaching agents can be used, however one of the most popular is hydrochloric acid, very often with the addition of oxidizing agent: HNO_3_, H_2_O_2_, Cl_2_, NaClO^19,24–28^. Hydrochloric acid is not only the most popular, but at the same time the only profitable leaching agent in which all precious metals can be dissolved and concentrated. In such a solution, platinum, palladium, rhodium and gold form complexes that can be concentrated and separated by ion exchange technique, like [PtCl_4_]^2−^, [PtCl_6_]^2−^, [Pt(OH)Cl_5_]^2−^, [Pt(OH)_2_Cl_4_]^2−^, [PdCl_4_]^2−^, [PdCl_6_]^2−^, [Pd(H_2_O)Cl_3_]^−^, [RhCl_6_]^3−^, [Rh(H_2_O)Cl_5_]^2−^, [Rh(H_2_O)_2_Cl_4_]^−^, [RhCl_6_]^2−^, [AuCl_2_]^−^, [AuCl_4_]^−^, [Au(OH)Cl_3_]^−^, [Au(OH)_2_Cl_2_]^−^^6,29–33^. The stability of precious metal chlorocomplexes depends on a large number of factors, including: redox potential, pH, free acid concentration and chloride ion concentration. For example the concentration of chloride ions largely influences the occurrence of given forms of chlorocomplexes. During dilution of a solution (which leads to a decrease in acidity), Pt(II) chloride complexes appear, which now coexist in various proportions with Pt(IV) chloride complexes, probably as a result of spontaneous reduction of Pt(IV) ions. However, that is not the sole occurrence—a range of aqua–chloro complexes may also form during dilution. Chloride complexes tend to form ion pairs with other functional groups according to the following series: [MCl_6_]^2–^ > [MCl_4_]^2–^ >> [MCl_6_]^3–^ > aqua complexes. Complexes with low charge densities pair more easily than those with higher charge densities, resulting in lower sorption efficiencies of aqua complexes due to their larger hydration shells. In a chloride environment, platinum, palladium, rhodium and gold occur mainly as anions, which is used in ion exchange^32,34^.

There are a lot of publications focusing on the recovery of precious metals using ion exchange method from chloride solutions^35^. In comparison to other techniques used for precious metals recovery, ion exchange method has low operating cost, low energy consumption, high selectivity, high efficiency, possibility of recovering metals from large volumes of low–concentration solutions, and uses simple equipment. Additionally, ion exchange resins can be regenerated and reused, resulting in a long service life of the bed and equipment^32,36^. However it is important to point out that most researches used synthetic solutions for their^37–44^, instead of an industrial one^45–48^. Using a technological solution often creates many problems, which are not appearing while using synthetic solutions. But that is not the only problem. Many publications focus only on tests in static conditions^38,39,45,47–50^, where the experiments are conducted in batch reactors. Many tests have to be redone in dynamic conditions, which show how the resin and solution would behave in conditions imitating industrial ones. In our previous work we checked the possibility of using three commercial ion exchange resins to recover precious metals from the technological solution obtained during the leaching of wastes of the refining processes. The experiments were conducted using a static method, to obtain knowledge about the process and understand how specific parameters influenced the efficiencies of metals sorption and elution. Parameters such as: volumetric ratio, process time, concentration of pollutants (Cu and Zn), concentration of HNO_3_, pH of the solution, temperature, concentration of thiourea and hydrochloric acid, were researched. In this article we carried out tests in dynamic conditions, to check the possibility of using the resins in industry and confirm the parameters which were previously researched and determined. We also calculate the operational and total capacity of the resins, which has not been done previously for industrial solutions in any publication for these resins^51,52^. It is worth mentioning that using solutions obtained after leaching of from refining waste has not been done previously and it may be a new alternative source of these metals for European Union.

The research conducted in this publication will hopefully help understand the problems of recycling of other types of wastes, which can be a source of precious metals for the EU. Additionally the research was conducted with a focus on possible industrial use.

## Materials and methods

### Materials

Puromet MTS9200 and Puromet MTS9850 were supplied by Purolite (King of Prussia, PA, USA), and Lewatit MonoPlus MP600 was provided by Lanxess Energizing Chemistry (Cologne, Germany). Their main characteristics are reported in Table 1^51,52^.

**Table 1:** Characteristic of ion exchange resins used in the experiments.

Name	Type	Functional group	Ionic form	Matrix	Moisture retention [%]	Particle size [µm]	Company
Puromet MTS9200	Weak base	Isothiouronium	H+	Polystyrenic crosslinked with divinylbenzene	48–54	300–1200	Purolite
Puromet MTS9850	Weak base	Polyamine	FB	Polyacrylic crosslinked with divinylbenzene	56–63	300–1200	Purolite
Lewatit MonoPlus MP600	Strong base	Quaternary ammonium type 2	Cl−	Polystyrenic	55–60	550–650	Lanxess

Concentrates and wastes (e.g., filters and precipitates) containing precious metals were provided by the Łukasiewicz Research Network–Institute of Non–Ferrous Metals (Łukasiewicz–IMN, Gliwice, Poland) as the sources of metals for the technological solution. Nitric acid (65%, AR, Avantor, Gliwice, Poland) was used to investigate the impact of HNO_3_ on the sorption process and for the preparation of the technological solution via leaching. Hydrogen peroxide (30%, AR, Avantor, Gliwice, Poland) was applied for the leaching of the materials containing precious metals. Hydrochloric acid (35–38%, AR, Avantor, Gliwice, Poland) was utilized in the leaching, conditioning, and elution experiments. Zinc(II) oxide (Avantor, Gliwice, Poland) and copper(II) chloride (Avantor, Gliwice, Poland) were used to assess the effect of pollutants on the sorption process. NaOH (Avantor, Gliwice, Poland) was used to investigate the impact of pH on the sorption process. Thiourea (AR, Avantor, Gliwice, Poland) was utilized in the elution experiments. Demineralised water (< 2 μS/cm, Łukasiewicz–IMN) was used in all the experiments. The materials utilized in the experiments in the dynamic conditions are consistent with those which had been used in the static conditions to ensure continuity. The results of the static conditions experiments are already published^51,52^.

The technological solution used in the research (belonging to the Łukasiewicz-IMN) was produced from the concentrates and precipitates obtained during the refining process of precious metals. Those materials were leached with a mixture of hydrochloric acid, hydrogen peroxide and nitric acid at 90 °C, which allowed to obtain the technological solution. The quantitative analysis of the solution is presented in Table 2^51,52^.

**Table 2:** Quantitative analysis of the solution used in the research.

Concentration of metals in the technological solution [mg/dm^3^]					
Pt	Pd	Rh	Au	As	Zn
67.4	60.1	21.5	4.4	3.3	15.9
Cu	K	Na	V	Ca	Fe
24.1	< 0.05	4.9	< 0.05	0.6	0.4

Besides the precious metals (such as platinum, palladium, rhodium and gold), the solution also contains significant amounts of copper and zinc, which are often found in the industrial solutions.

Glass columns with an internal diameter of 1.5 cm and a height of 25 cm were used for the tests. They were equipped with: glass cups with an internal diameter of 2.5 cm and a height of 10 cm, and a bottom made of a G1 sinter.

### Methods

Before any experiment was conducted the resins were conditioned with 10% (w/w) HCl. This stage was carried out to make sure that all the functional groups were activated and therefore were able to exchange ions. This step was conducted according to the description in the previous publication^51^.

Experiments were performed to determine the influence of contact time (between the technological solution and the bed in the column) on the course of the sorption process. For this purpose, the technological solution (V_s_ = 50 cm^3^) was passed through the bed of conditioned ion exchange resin (V_r_ = 5 cm^3^), placed in the column, at the ratio V_r_:V_s_ = 1:10, maintaining the specified flow rates (10 cm^3^/min, 5 cm^3^/min, 3.33 cm^3^/min, 1.67 cm^3^/min, 0.83 cm^3^/min, which correspond to contact times between the resin bed and solution = 5 min, 10 min, 15 min, 30 min, 60 min). After the set time, the resins were removed from the column, the volumes of the solutions and the resins were measured, and the concentrations of platinum, palladium, rhodium and gold in the solutions, as well as copper and zinc in selected samples, were determined.

Experiments were performed to determine the influence of bed geometry (the ratio of the bed height—h—to the internal diameter of the column—d) on the course of the sorption process. For this purpose, the technological solution was passed through the bed of conditioned ion exchange resin, placed in the column at the ratio V_r_:V_s_ = 1:10, maintaining the flow rate of 10 cm^3^/min (contact time of 5 min). The height of the bed was changed by introducing different volumes of resin into the column (V_r_ = 5; 7.5; 10; 15 and 20 cm^3^), and the amount of solution passed through the column was increased proportionally to the increasing volume of the bed (V_s_ = 50, 75, 100, 150 and 200 cm^3^). After the set time, the resins were removed from the column, the volumes of the solutions and the resins were measured, and the concentrations of platinum, palladium, rhodium and gold in the solution were determined.

Experiments were performed to determine the effect of the concentration of nitric acid in the feed on the course of the sorption process. The initial concentration of nitric acid was 0.52 g/dm^3^, and the additional tested concentrations were 70 g/dm^3^ and 150 g/dm^3^. The measured theoretical volume of the concentrated nitric acid was mixed with the technological solution, obtaining a solution containing the set amount of precious metals and nitric acid. Then, the technological solution (V_s_ = 50 cm^3^) was passed through the bed of the conditioned ion exchange resin placed in the column (V_r_ = 5 cm^3^), at the ratio V_r_:V_s_ = 1:10, maintaining the flow rate of 10 cm^3^/min (contact time of 5 min). After the set time, the resins were removed from the column, the volumes of the solutions and the resins were measured, and the concentrations of platinum, palladium, rhodium and gold in the solution were determined.

Experiments were performed to determine the influence of the pH of the technological solution on the sorption process. To achieve the appropriate pH, NaOH was added to the technological solution until the pH meter showed the appropriate value. Then, the technological solution (V_s_ = 50 cm^3^) was passed through the bed of the conditioned ion exchange resin placed in the column (V_r_ = 5 cm^3^), at the ratio V_r_:V_s_ = 1:10, maintaining the flow rate of 10 cm^3^/min (contact time of 5 min). After the set time, the resins were removed from the column, the volumes of the solutions and the resins were measured, and the concentrations of platinum, palladium, rhodium and gold in the solution were determined.

Experiments were performed to determine the influence of the copper concentration on the sorption process. The calculated theoretical amount of copper(II) chloride was dissolved in the technological solution, obtaining the target solution containing the appropriate concentration of precious metals and copper. Then, the technological solution (V_s_ = 50 cm^3^) was passed through the bed of the conditioned ion exchange resin placed in the column (V_r_ = 5 cm^3^), at the ratio V_r_:V_s_ = 1:10, maintaining the flow rate of 10 cm^3^/min (contact time of 5 min). After the set time, the resins were removed from the column, the volumes of the solutions and the resins were measured, and the concentrations of platinum, palladium, rhodium and gold in the solution were determined.

Experiments were performed to determine the influence of the zinc concentration on the sorption process. The calculated theoretical amount of zinc(II) oxide was dissolved in the technological solution, obtaining the target solution containing the appropriate concentration of precious metals and zinc. Then, the technological solution (V_s_ = 50 cm^3^) was passed through the bed of the conditioned ion exchange resin placed in the column (V_r_ = 5 cm^3^), at the ratio V_r_:V_s_ = 1:10, maintaining the flow rate of 10 cm^3^/min (contact time of 5 min). After the set time, the resins were removed from the column, the volumes of the solutions and the resins were measured, and the concentrations of platinum, palladium, rhodium and gold in the solution were determined.

Experiments were performed to determine the influence of the contact time of the eluent with the bed on the course of the elution process. For this purpose, a solution of 2 mol/dm^3^ thiourea in 1 mol/dm^3^ hydrochloric acid (V_s_ = 50 cm^3^) was passed through the bed of the after-sorption resin placed in the column (V_r_ = 5 cm^3^), at the ratio V_r_:V_s_ = 1:10, maintaining the specified flow rates (10 cm^3^/min, 5 cm^3^/min, 3.33 cm^3^/min, 1.67 cm^3^/min, 0.83 cm^3^/min, which correspond to contact times between the resin bed and solution = 5 min, 10 min, 15 min, 30 min, 60 min). After the set time, the resins were removed from the column, the volumes of the solutions and the resins were measured, and the concentrations of platinum, palladium, rhodium and gold in the solution were determined.

Experiments were also performed to determine the influence of the number of cycles on the sorption and elution process, and the stability of selected resins. Five cycles of sorption (V_r_ = 5 cm^3^, V_s_ = 50 cm^3^, t_contact_ = 5 min—flow rate = 10 cm^3^/min) and elution (V_s_ = 50 cm^3^ 2 mol/dm^3^ thiourea in 1 mol/dm^3^ HCl, t_contact_ = 5 min—flow rate = 10 cm^3^/min) were performed alternately. Between the sorption and elution steps, the bed in the column was washed with demineralized water until the pH of the effluent was approximately 4. After the last cycle, the resins were removed from the column, the volumes of the solutions and the resins were measured, and the concentrations of platinum, palladium, rhodium and gold in the solution were determined.

Experiments were performed to determine the operating capacity and total capacity of the ion exchange resins. For this purpose, the technological solution was passed through 5 cm^3^ of the conditioned bed placed in the column, maintaining the flow rate of 10 cm^3^/min (contact time of 5 min). These tests were performed throughout the day, then the bed was left in water overnight after washing it to the pH of the leakage of approximately 4, and the process was repeated the next day. Samples of the after-sorption solution were collected every 20 bed volumes (BV, 100 cm^3^) and analysed for the content of platinum, palladium, rhodium and gold.

### Analytical methods

The analyses were conducted at the Centre of Analytical Chemistry (Łukasiewicz Research Network – Institute of Non–Ferrous Metals, Gliwice, Poland). The concentrations of platinum, palladium, rhodium, and gold in the solution samples were determined using Inductively Coupled Plasma Mass Spectrometry (ICP-MS; NexION 300D, PerkinElmer, Waltham, MA, USA). Copper and zinc were analysed using Flame Atomic Absorption Spectroscopy (FAAS; SOLAAR S4, Thermo, Waltham, MA, USA). The microscopy photos were taken in the Centre of Advanced Materials Technologies using a VHX-7000 digital microscope (Keyence, Mechelen, Belgium), using 100× magnification. To analyse the composition of the ion-exchange resins a ZSX Primus WDXRF (Rigaku, Tokyo, Japan) was used. The average error of the method was between 10 and 20%, depending on the dilution of the samples and the concentration of precious metals. The analytical methods used in the experiments in the dynamic conditions are consistent with those which had been used in the static conditions to ensure continuity. The results of the static conditions experiments are already published^51,52^.

### Calculations

The metals sorption efficiencies (SE, %) were calculated according to the formula:

1 $$ {\text{SE}} = \frac{{{\text{C}}_{{\text{i}}} - {\text{C}}_{{\text{f}}} }}{{{\text{C}}_{{\text{i}}} }} \times 100\% $$

C_i_: initial concentration of the metal in the solution, mg/dm^3^; C_f_: final concentration of the metal in the solution, mg/dm^3^.

The metals elution efficiencies (E, %) were calculated according to the formula:

2 $$ {\text{E}} = \frac{{{\text{m}}_{{\text{f}}} }}{{{\text{m}}_{{\text{i}}} }} \times 100\% $$

m_i_: initial amount of metal in the resin sample, mg; m_f_: final amount of metal in the eluate sample, mg.

The degrees of saturation of the ion–exchange resins with a given metal (SN_M_, mg_metal_/cm^3^_ion–exchange resin_) were calculated according to the formula:

3 $$ SN_{M} = \frac{{m_{M} }}{{V_{r} }} $$

m_M_: mass of a metal sorbed on the ion–exchange until the operating or total capacity is reached, mg; V_r_: volume of resin used in the sorption process, cm^3^.

## Results and discussion

**Figure 1:**
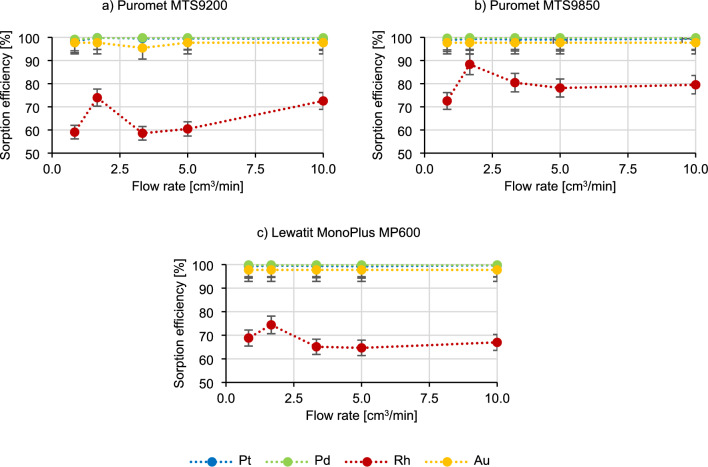
Dependence of the flow rate on the metal sorption efficiency.

The initial step in performing experiments under dynamic conditions involves verifying the contact time between a bed and a solution. These dynamic conditions simulate industrial settings, making it advantageous to explore the feasibility of shortening the process duration. Given the large scale of production in various industrial sites, it is often essential for a resin to efficiently adsorb elements within a brief timeframe. In the Fig. 1 the results of the impact of contact time on the course of the sorption process are present using three different resins.

Based on the results in Fig. 1, it can be said that the sorption efficiency of platinum, palladium and gold does not change with increasing the flow rate of the technological solution. Rhodium behaves most characteristically—in the beginning the sorption efficiency increases (for example, in the case of Puromet MTS9200 from 59.1% to 74.0%), then decreases rapidly (from 74.0% to 58.6% for Puromet MTS9200), which again has an increasing tendency again later on (to 72.6% for Puromet MTS9200). Picking the optimal flow rate in the industrial conditions is contingent on various interconnected processes. It is possible that sorption efficiency may improve at higher flow rates, however maintaining an excessively high flow rate is impractical. This is due to several factors, including the interplay between different processes that either supply materials for sorption or utilize them in the industry. Another consideration is equipment efficiency, as running equipment at maximum speed can result in higher energy costs. Moreover, the flow resistance of ion-exchange resins also poses a challenge, as over time, resins degradation and destruction increases flow resistance. Consequently, passing the solution through the bed at a higher flow rate could lead to significantly higher energy and operational costs, which is not optimal for industrial operations. Therefore, due to the high sorption efficiency of precious metals at the flow rate of 10 cm^3^/min (contact time 5 min), it was chosen as the basic parameter for further research.

**Figure 2:**
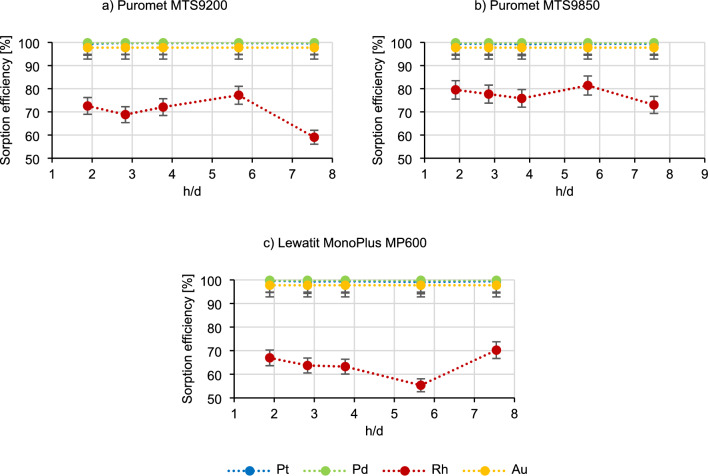
Dependence of the bed geometry (h/d) on the metal sorption efficiency.

Another critical factor to assess under dynamic conditions is the bed geometry. Several resins may undergo volume changes in acidic or basic solutions, potentially impacting their operational efficiency. Therefore, it is essential to consider the optimal bed geometry when designing columns for future industrial applications. In the Fig. 2 the results of the impact of the bed geometry on the course of the sorption process are present.

The sorption efficiency of platinum, palladium and gold does not change with the difference in h/d, judging by the results presented in Fig. 2. The most specific obtained results are once again for rhodium, for which the sorption efficiency in the case of the Puromet MTS9200 remains at a similar level (about 70%), and after exceeding h/d ~ 6 it sharply drops (to 59.1%), in the case of using Puromet MTS9850, it remains at a similar level (about 76%), and in the case of using Lewatit MonoPlus, it first decreases with increasing h/d (from 67.0% to 55.3%), and after exceeding h /d ~ 6 increases rapidly (up to 70.2%). However, at the current level of research it is not possible to draw any specific conclusions and it can be assumed that the bed geometry does not influence the sorption efficiencies.

In the subsequent experiments, the impact of various factors (such as nitric acid, copper, and zinc concentration, and solution pH) on sorption efficiencies was investigated. These factors had been previously examined in static conditions, but it was deemed necessary to also evaluate them under dynamic conditions. As a result, only specific data points were retested in dynamic conditions to verify if the observed trends remained consistent^52^.

**Figure 3:**
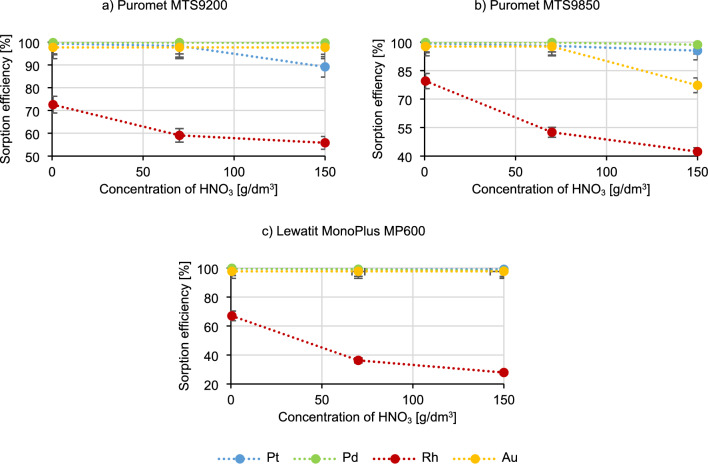
Dependence of the metal sorption efficiency on the concentration of nitric acid.

Figure 3 shows the results of the impact of the nitric acid concentration on the course of the sorption process.

Data included in Fig. 3 presents that as the concentration of nitric acid increases, the rhodium sorption efficiency decreases, in the case of Puromet MTS9200 to a level of approximately 55%, in the case of Puromet MTS9850 to 40%, and in the case of Lewatit MonoPlus MP600 to 30%. As the concentration of nitric acid increases, the platinum sorption efficiency for Puromet MTS9200 also decreases (from 98.4% to 89.2%), as for Puromet MTS9850 the gold sorption efficiency (from 97.7% to 77.3%) and also to a small extent platinum and palladium (change from 98.1% to 95.5% for Pt and from 99.8% to 98.7% for Pd). The visible decrease in the precious metals sorption efficiencies in the case of using Puromet MTS9850 is probably caused by the gel nature of this resin, which can degrade under the influence of the oxidizing conditions of nitric acid. Additionally, very acidic environment is not suitable for many resins due to the possibility of oxidation of functional groups.

**Figure 4:**
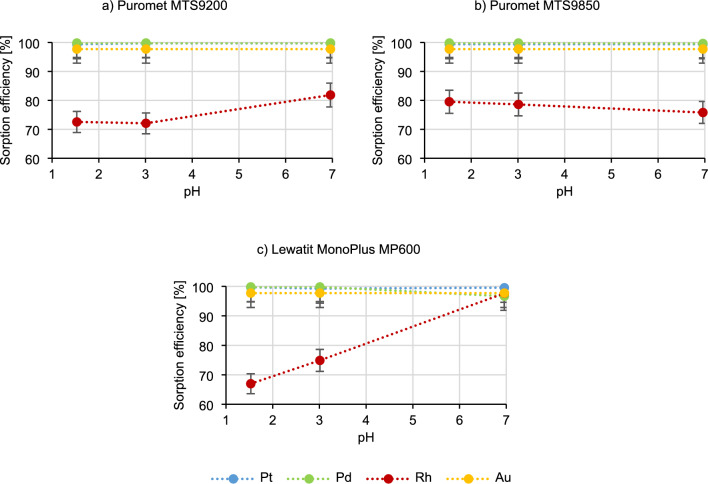
Dependence of the metal sorption efficiency on the pH of the technological solution.

Figure 4 shows the results of tests on the influence of the pH of the technological solution on the sorption process.

In the case of Puromet MTS9200 and Puromet MTS9850, the sorption efficiency of platinum, palladium and gold does not change with the increasing pH. The rhodium sorption efficiency using Puromet MTS9200 increases (from 72.6% to 81.9%), and in the case of Puromet MTS9850 it decreases (from 79.5% to 75.8%). Distinctive results are observed using Lewatit MonoPlus MP600, where during the pH increase, the palladium sorption efficiency decreases (from 99.8% to 96.7%), but the rhodium sorption efficiency increases rapidly, reaching results even above 97%—at the pH of approximately 7. The same situation occurred when this parameter was tested in the static conditions. However why such situation occurs only while using one resin? The first explanation can be rhodium behaviour in the solutions with the increased pH. According to^53^ rhodium in the solutions with the pH above 2.9 undergoes hydrolysis according to a formula:

$$ \left[ {{\text{RhCl}}_{{{6} - {\text{n}}}} \left( {{\text{H}}_{{2}} {\text{O}}} \right)_{{\text{n}}} } \right]^{{{\text{n}} - {3}}} \to \, \left[ {{\text{RhCl}}_{{{6} - {\text{n}}}} \left( {{\text{H}}_{{2}} {\text{O}}} \right)_{{{\text{n}} - {1}}} {\text{OH}}} \right]^{{{\text{n}} - {1} - {3}}} + {\text{ H}}^{ + } $$

The new complex may have higher affinity towards the functional groups of the Lewatit MonoPlus MP600 resin (Quaternary ammonium type 2). Although it does not fully explain why such occurrence can be only observed for one resin. Among the three studied resins, Lewatit MonoPlus MP600 is the only one with strong base groups. According to^34^ it can be concluded that the distribution of Rh(III) drops significantly with the increase of chloride ion concentrations. Therefore when NaOH is added, Na^+^ ion combines with Cl^-^ ion, creating a new stable compound. The concentration of free chloride ions in the solution drops and the distribution coefficient for Rh(III) increases. In weak base resins (Puromet MTS9200 and Puromet MTS9850) the distribution coefficient for rhodium is not influenced by the free chloride ion concentration. This means that to recover rhodium from technological solutions, Lewatit MonoPlus MP600 can be used, after prior neutralization of the solution.

**Figure 5:**
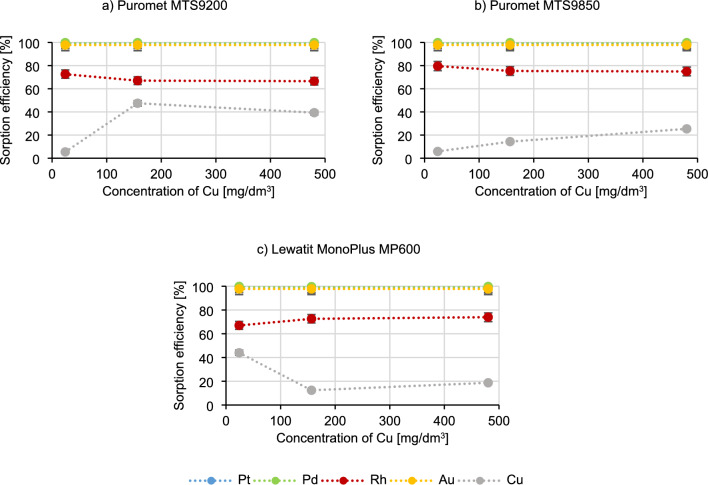
Dependence of the metal sorption efficiency on copper concentration.

Figure 5 presents the results of researching the influence of copper concentration on the sorption process.

The graphs in Fig. 5 show that the sorption efficiencies of platinum, palladium and gold do not change with increasing copper concentration. When using Puromet MTS9200 and Puromet MTS9850, with an increase in copper concentration, its sorption efficiency also increases (for Puromet MTS9200 from 5.4% to 47.4%, and for Puromet MTS9850 from 5.8% to 25.3%) and at the same time the rhodium sorption efficiency decreases (by approximately 5% for both resins). A completely opposite situation occurs when Lewatit MonoPlus MP600 is used, where the copper sorption efficiency decreases (from 44.0% to 12.4%) and rhodium sorption efficiency increases (from 67.0% to 74.0%). This means that Puromet MTS9200 should not be used for solutions containing a high concentration of copper, because the copper ion may become a competitive ion in relation to the precious metal ions. Such situation is definitely problematic while dealing with precious metals solutions which often contain copper. According to^54^ copper belongs to a group of metals which exist in the form of equilibrating anionic species, therefore their behaviour is hard to predict:

$$ {\text{CuCl}}\left( {{\text{H}}_{{2}} {\text{O}}} \right)_{{3}}^{ + } \leftrightarrow {\text{ CuCl}}_{{2}} \left( {{\text{H}}_{{2}} {\text{O}}} \right)_{{2}} \leftrightarrow {\text{ CuCl}}_{{4}}^{{{2} - }} $$

**Figure 6:**
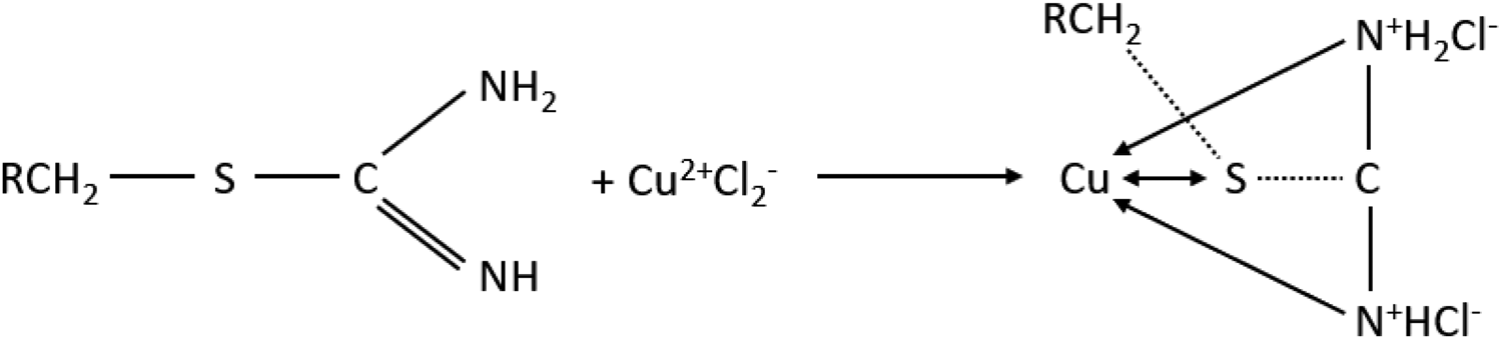
Schematic reaction of the formation of copper complex with Puromet MTS9200.

**Figure 7:**
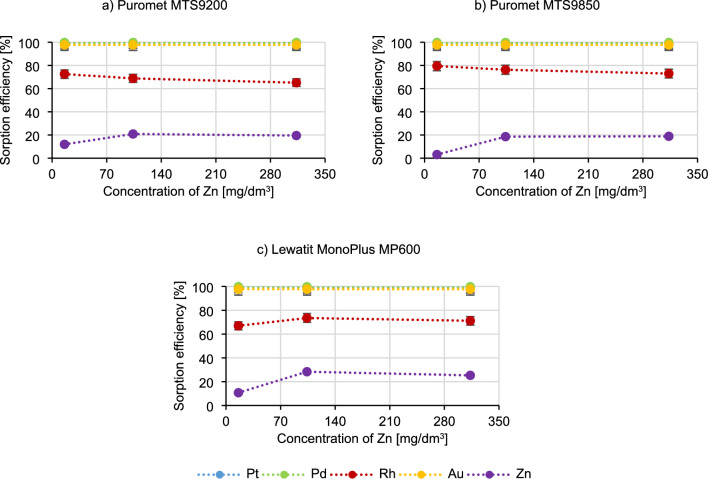
Dependence of the metal sorption efficiency on zinc concentration.

Perhaps while adding Cu(II), more chloride complexes of Cu(II) are being formed which are competing with Pd(II) ions. Additionally Puromet MTS9200 is the only resin which contains isothiourionium functional groups—perhaps in the solutions with the higher concentrations of both copper and chloride ion (which was added with copper), Cu is starting to get higher affinity in comparison to the rest of precious metals as the stability constants of Cu–Cl complexes are lower than those of precious metals chlorocomplexes^55^. Additionally, copper has a high affinity for sulphur, which is also an important factor in the formation of the isothiuronium complex with Cu. In Fig. 6 a probable schematic reaction of the formation of Cu complex is presented^56^:

Figure 7 presents the results of testing the influence of zinc concentration on the sorption process.

Similarly, to the case of copper, when the zinc concentration increases, the sorption efficiency of metals such as platinum, palladium and gold does not change. The rhodium sorption efficiency when using Puromet MTS9200 and Puromet MTS9850 decreases, but only slightly (by about 6%), and in the case of Lewatit MonoPlus MP600, it can be said that the sorption efficiency practically does not change. The sorption efficiency of zinc increases as its concentration raises, but in all the tested conditions it did not exceed 30%. Based on the results, it can be concluded that Lewatit MonoPlus MP600 is not recommended for use for solutions containing high concentrations of zinc, because it may become a competitive ion towards precious metal ions. In the case of Zn the situation may be similar to Cu^54^. This metal is also in equilibrium with either neutral or cationic complexes:

$$ {\text{ZnCl}}\left( {{\text{H}}_{{2}} {\text{O}}} \right)_{{\text{x}}}^{ + } \, \leftrightarrow \,{\text{ZnCl}}_{{3}} \left( {{\text{H}}_{{2}} {\text{O}}} \right)_{{\text{y}}}^{ - } \, \leftrightarrow \,{\text{ZnCl}}_{{4}}^{ - } $$

Therefore, the same rationale used for copper can be applied to zinc as well. In addition, similar tests were conducted in a publication researching the sorption of platinum from the solutions containing Zn, Al, Fe, Cu and Ni. According to this article, [ZnCl_4_]^2−^ is a competitive ion to [PtCl_6_]^2−^ in the Pt microcomponent–ZnCl_2_ macrocomponent system, which is a situation which also is happening here, while adding additional amounts of Zn^57^.

**Figure 8:**
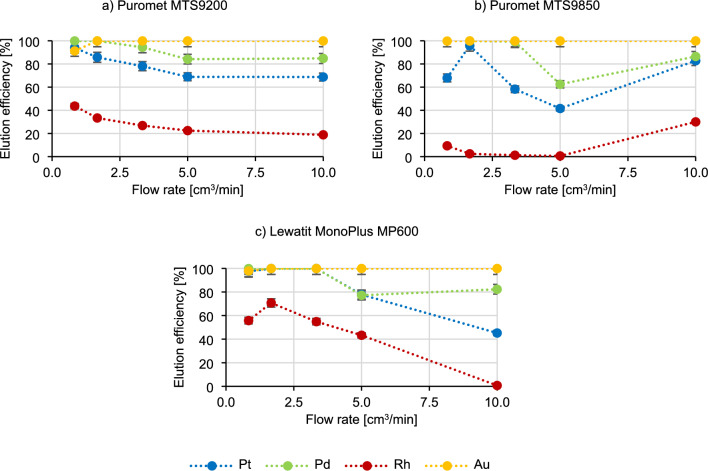
Dependence of the metal elution efficiency on flow rate.

In the previous static conditions tests, the impact of various parameters of the elution process on metal elution efficiencies was investigated. A solution containing 2 mol/dm^3^ of thiourea in 1 mol/dm^3^ of HCl was identified as the most effective eluting agent, with a volumetric ratio of V_r_:V_e_ = 1:10 (V_e_—volume of the eluting agent) deemed the most optimal. In the dynamic conditions these were used as the basic parameters to conduct the experiments. The influence of the contact time of the eluent with the bed on the elution process was researched and the results are presented in Fig. 8.

Based on the graphs in the Fig. 8, it can be deduced that gold elutes at a consistently high level (> 90%) at every flow rate. In the case of Puromet MTS9200, as the flow rate increases, the elution efficiency of each precious metal decreases (for Pt from 93.9% to 68.7%; for Pd from 99.9% to 84.1%; and for Rh from 43.7% to 18.9%). The same situation is visible for Lewatit MonoPlus MP600 (for Pt from 99.9% to 45.2%; for Pd from 99.9% to 82.3% to; and for Rh from 70.7% to 0.7%). In case of Puromet MTS9850 an decreasing tendency of elution efficiency of Pd (from to 99.8% to 83.4%) and Pt (from 99.9% to 41.6%) is visible. The elution of Rh is almost impossible while using Puromet MTS9850, although this metal can be later on recovered from the resin after elution by burning it in specials furnaces. This way obtaining concentrate containing only Rh is possible. The optimal elution results for each precious metal are obtained with the flow rates ranging from 1.67 to 5.00 cm^3^/min.

All experiments showed that rhodium was the most difficult element to recover due to its kinetically inert nature, which results in the largest fluctuation in its sorption efficiency^58^. As already mentioned, in acidic solutions, especially in aqueous chloride solutions, rhodium forms many complexes, such as [RhCl_6_]^3−^, [Rh(H_2_O)Cl_5_]^2−^, [Rh(H_2_O)_2_Cl_4_]^−^ or [RhCl_6_]^2−^^30,59^. Although rhodium can occur in fourth oxidation state, it more often appears in chloride solutions in its third oxidation state, and undergoes solvation. This means that compounds with the H_2_O or OH ligands are formed. Precious metal complexes have a specific, already mentioned, tendency to form ion pairs with ion–exchange resin’s functional groups, according to the following series: [MCl_6_]^2−^ > [MCl_4_]^2−^ >> [MCl_6_]^3−^ > aqua complexes. It shows that rhodium in the form of aqua complexes in the third oxidation state is the last to undergo the ion exchange process, which explains its low sorption efficiency and therefore largest fluctuation in its sorption efficiency.

**Figure 9:**
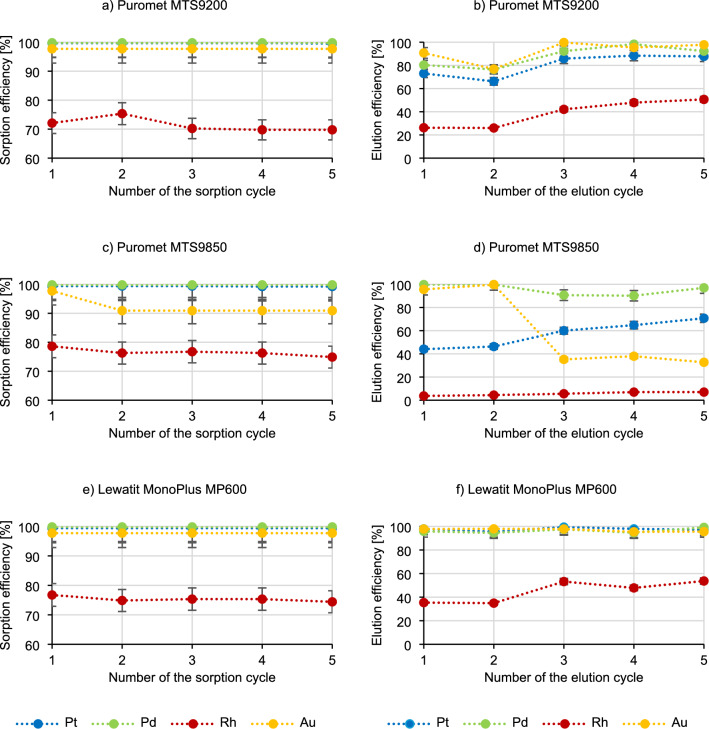
The dependence of the sorption and elution efficiency on the cycle number.

Figure 9 presents the results of five sorption–elution cycles.

Based on the analysis of the results presented in Fig. 9, it is visible that in the case of sorption using Puromet MTS9200, the sorption efficiency of precious metals remains at the same level. Using the same resin, the elution efficiency of precious metals increases with each successive elution cycle. In the case of sorption using Puromet MTS9850, there is a visible downward trend in the gold sorption efficiency (from 97.7% to 90.9%, already in the second cycle) and a low decrease in the sorption efficiency of rhodium and platinum (by 1–5%). In the elution process using this resin, there is also a visible decrease in the elution efficiency of gold (from 95.6% to 32.7%) and an increase in the elution efficiency of platinum (from 44.1% to 70.7%). In the case of Lewatit MonoPlus MP600, the efficiency of precious metals in the sorption process remains at a similar level as the number of cycles increases. Similarly, in the elution process, there is no change in the elution efficiency for platinum, palladium, and gold, and only increases for rhodium (from 35.4% to 53.7%).

**Figure 10:**
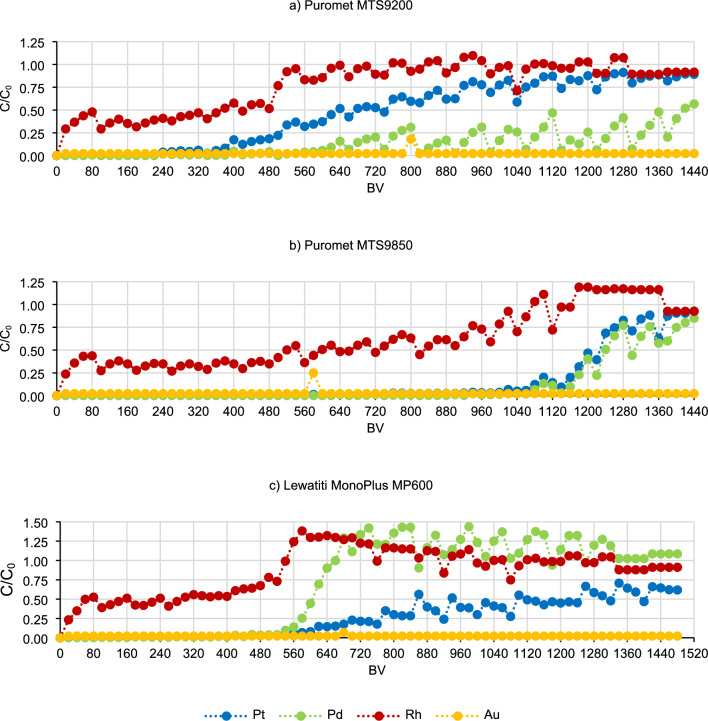
Sorption curves.

Subsequently, continuous sorption tests were carried out to explore the potential for achieving operational and total capacity of the resins. This investigation aimed to provide insights into the conditions under which ion exchange resins can operate most efficiently. The results of the continuous tests are presented in Fig. 10.

**Figure 11:**
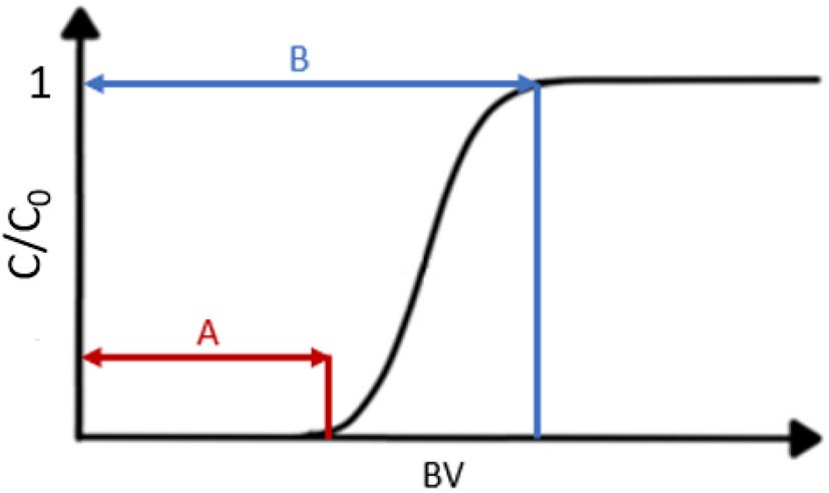
Theoretical sorption curve (C—final concentration of metal in the post-sorption solution, C_0_—initial concentration of metal in the pre-sorption solution).

The obtained graphical curves indicate that the sorption process can be carried out for an even longer time, because not all points at which the operating and total capacity can be determined have been reached. However, based on the obtained results, the operating capacity for selected metals can be calculated—it is the first inflection point when the concentration of metal in the post-sorption solution begins to increase rapidly (line A in Fig. 11). The period before reaching the operating point determines the best working capacity of the column. In some cases, it was also possible to determine the total capacity—it is the point at which the metal concentration in the post-sorption solution equals the concentration in the pre-sorption solution dosed to the column (line B in Fig. 11). This is the moment when the resin will no longer adsorb the metal.

For specific resins, the operating and total capacities and their corresponding degrees of saturation were determined and summed in Table 3. The concentrations of metals in the post-sorption solutions after continuous sorption tests are presented in Tables SD-1–SD-3 in Supplementary Data. The data was utilized for plotting the curves, so all subsequent conclusions also pertain to the data presented in the Supplementary Data Tables.

**Table 3:** Results of the continuous sorption tests.

Ion-exchange resin	Metal	Operating capacity		Total capacity	
		[BV]	Degree of saturation [mg_metal_/cm^3^_resin_]	[BV]	Degree of saturation [mg_metal_/cm^3^_resin_]
Puromet MTS9200	Pt	340	22.77	1260	51.14
	Pd	600	36.36	–	–
	Rh	480	6.02	520	6.16
Puromet MTS9850	Pt	1060	74.18	1400	85.32
	Pd	1060	67.12	–	–
	Rh	980	12.20	1060	12.54
Lewatit MonoPlus MP600	Pt	560	36.32	–	–
	Pd	540	31.26	660	34.53
	Rh	520	5.28	540	5.28

Operating and total capacities are very important parameters while trying to scale up the process in dynamic conditions. As previously mentioned, operating capacity is the range when the column is working with the best efficiencies. In the industry, when this point is overpassed, the solution is then dosed to another column and the first one undergoes elution and regeneration step. In Table 3 it can be seen that the highest operating capacity was obtained for Puromet MTS9850 (for Pt and Pd 1060 BV, for Rh 980 BV), therefore in the industrial conditions this resin would be able to work for a longer time during one cycle than the other two tested resins. The operating capacities for different metals while using both Puromet MTS9850 and Lewatit MonoPlus MP600 are pretty similar, which allows to stop the process in one moment. The situation looks different while using Puromet MTS9200—it is almost impossible to assign one point in which the sorption should be stopped. In multicomponent solutions, especially while trying to recover more than one metal, it is better if most of the elements have similar operating capacities. The total capacity is a point when the resin cannot sorb more of the specific metal. For these tests, the threshold for reaching the total capacity was set at 0.9 instead of 1, taking into account the low levels of precious metals and potential analytical measurement errors of up to 10%. It is very important to mention that the total capacity and the ion-exchange capacity (in this case anion-exchange capacity) are not the same thing. Total capacity can be calculated for a specific solution and it will differ while using different one, while the ion-exchange capacity is the maximal amount of ions which can be sorbed by the resin.

In the case of gold, neither the operating capacity nor the total capacity could be achieved using these three ion exchange resins. The reason for this is probably the low concentration of gold in the initial solution and the high affinity of it to the ion exchange resins.

Microscopy photos of the ion exchange resins were also taken before conditioning and after continuous sorption experiments. The photos are shown in Fig. 11.

**Figure 12:**
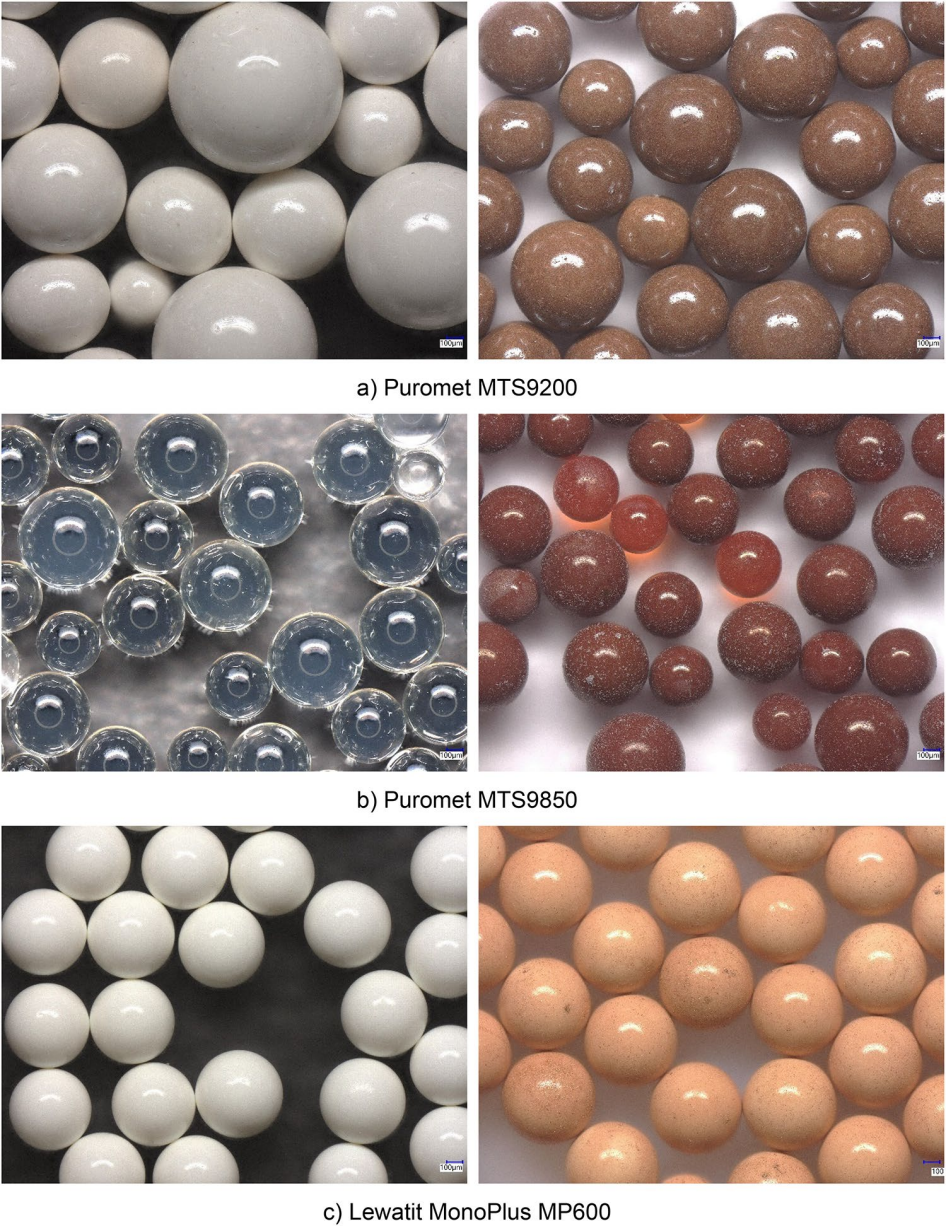
Microscopy photos of the ion exchange resins (on the left—before the process, on the right—after continuous sorption experiments).

As can be seen in Fig. 12, Puromet MTS9200 contains beads of the most diverse sizes, while Lewatit MonoPlus MP600’s beads have very similar dimensions. A change in the colour of the ion-exchange resins after the sorption process is also visible, probably due to the sorption of precious metal complexes of different colours (from red to yellow). In no case were any significant defects such as chips, cracks or fractures observed.

In Table 4 a semi-quantitative analysis of the ion-exchange resin, before and after the sorption is presented.

**Table 4:** Semi-quantitative analysis of the ion–exchange resins before and after sorption.

Element	Puromet MTS9200		Puromet MTS9850		Lewatit MonoPlus MP600	
	Content before sorption [%]	Content after sorption [%]	Content before sorption [%]	Content after sorption [%]	Content before sorption [%]	Content after sorption [%]
C	54.40	45.20	48.60	30.90	52.20	42.70
Cl	22.20	9.02	0.18	5.70	36.30	27.40
O	3.47	5.08	50.40	30.20	11.40	3.99
Fe	0.04	0.06	0.16	0.04	0.04	0.01
Si	0.02	0.04	0.14	0.02	0.02	0.02
Ca	0.01	0.02	0.23	0.02	0.01	0.02
S	19.8	10.00	0.07	0.07	–	0.09
Pt	–	11.30	–	14.10	–	19.5
Pd	–	15.70	–	13.40	–	4.96
Rh	–	1.46	–	2.53	–	0.48
Au	–	1.39	–	1.53	–	0.70

The structure of ion-exchange resins such as Puromet MTS9850 and Lewatit MonoPlus MP600, which are composed of polystyrene crosslinked with divinylbenzene, was confirmed through semi-quantitative analysis data. These resins mainly consisted of carbon, with the incorporation of chlorine indicating the presence of vinyl chloride or a chloride radical added in the polymerization process. Puromet MTS98500, on the other hand, has a matrix of polyacrylate crosslinked with divinylbenzene, as shown by the high carbon and oxygen content. Despite undergoing sorption processes, the composition of ion-exchange resins remains largely unchanged, with any variations in elemental content attributed to the proportional appearance of precious metals.

It is important to note that there are currently no published studies addressing the calculation of resins capacities in dynamic conditions using industrial solutions. Only one publication describes the research conducted in dynamic conditions while using one of the same ion-exchange resins (Puromet MTS9850)^46^. The comparison is included in Table 5.

**Table 5:** Comparison of the research results with literature data.

Parameter	Literature^46^	This publication
Ion-exchange resins	Purolite S985, Purolite A500, AM-2B	Puromet MTS9850, Puromet MTS9200, Lewatit MonoPlus MP600
Type of the solution	Synthetic (containing Pt and Rh)	Industrial (containing Pt, Pd, Rh and Au)
Sorption results	For Purolite S985 Pt 90.7–99.9% Rh 39.7–99.9%	For Purolite S985 Pt > 99.8% Pd > 99.8% Rh 72.6–88.4% Au > 97.7%
Elution results	For Purolite S985 Pt 2 mol/dm^3^ HCl: 95–99% Pt 2 mol/dm^3^ NH_4_SCN: 94–99% Rh 2 mol/dm^3^ HCl: > 88%	For Puromet MTS9850 Pt 2 mol/dm^3^ thiourea in 1 mol/dm^3^: 41.6–99.9% Pd 2 mol/dm^3^ thiourea in 1 mol/dm^3^: 62.5–99.9% Rh 2 mol/dm^3^ thiourea in 1 mol/dm^3^: 0.6–30.0% Au 2 mol/dm^3^ thiourea in 1 mol/dm^3^: > 99.9%

This research represents a novel approach in the scientific community for recovering precious metals through ion-exchange method using waste of the refining process. It marks a significant advancement towards industrial application and will greatly contribute to scaling the process in future studies.

Conducting a full economic study may be impossible at this point of research as the full technology for the production of pure precious metals from the refining waste has not been yet developed. However for comparative purposes, we can assume that at the beginning the possible installation can process 1 tonne of refining waste containing 0.05% of each precious metal (Pt, Pd, Rh, and Au). The recovery of precious metals is never complete, which is why the efficiency of the entire technology was assumed to be 99%. The market prices of individual precious metals as of May 13, 2024 (according to kitco.com^60^) were: for gold—EUR 69,825.42/kg; for platinum—EUR 29,721.25/kg; for palladium—EUR 28,975.98/kg; for rhodium—EUR 137,099.32/kg. This means that the installation can produce precious metals (with a purity of 99.9%) worth: 0.495 kg of gold—EUR 34,563.58; 0.495 kg of platinum—EUR 14,712.02; 0.495 kg for palladium—EUR 14,343.11; 0.495 kg for rhodium—EUR 67,864.16. Therefore, from 1 tonne of low-quality waste the possible installation can manufacture precious metals worth EUR 131,482.87.

## Conclusion

**Figure 13:**
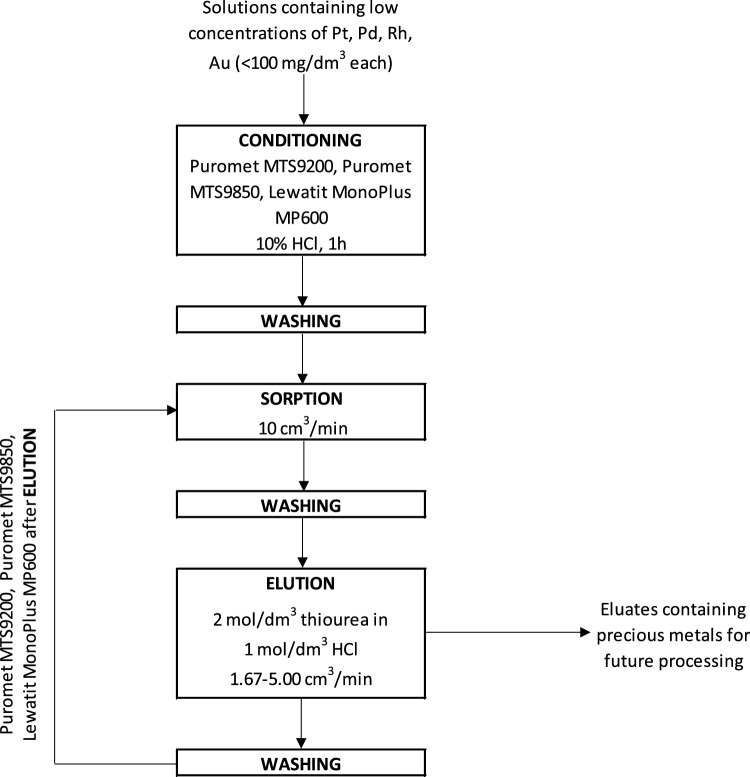
Process diagram.

The research findings indicated that ion-exchange resins, namely Puromet MTS9200, Puromet MTS9850, and Lewatit MonoPlus MP600, have the potential to recover precious metals (platinum, palladium, rhodium, and gold) from the solution generated after the leaching of the refining process wastes. Various factors influencing the sorption in the dynamic conditions were identified. The contact time between the solution and the resin bed should be 5 min. It was observed and confirmed that an increase in the concentration of elements like copper and zinc could impact the sorption process, as these metals may compete with the precious metals for the active sorption sites. It is not recommended to use Puromet MTS9850 for solutions with high nitric acid concentration, as the sorption efficiencies of platinum, palladium, rhodium, and gold are decreasing. To enhance the sorption efficiency of rhodium, it is advisable to neutralize the acidic solution before the process and use Lewatit MonoPlus MP600, which enables the recovery of rhodium with the sorption efficiency exceeding 90%. The most effective eluent for recovering precious metals from the resins is a solution consisting of 2 mol/dm^3^ thiourea in 1 mol/dm^3^ hydrochloric acid with the flow rate between 1.67 and 5.00 cm^3^/min. The scheme of the ion exchange process is presented in Fig. 13.

The results obtained in this research will help conduct further research regarding the production of materials containing precious metals from eluates.

### Supplementary Information

Supplementary Information.

## Data Availability

The authors confirm that the data supporting the findings of this study are available within the article and its supplementary materials. Additional data that support the findings of this study are available from the corresponding author (Karolina Goc) upon reasonable request.
