# Supplementary material for: Application of ion-exchange dynamic conditions in the recovery of precious metals from refining waste
Source: Sci Rep. 2024 Jul 1;14:15026. doi: 10.1038/s41598-024-66086-x (PMC11217456; doi:10.1038/s41598-024-66086-x)
Supplement: Supplementary file 1 — Supplementary Information. [file 41598_2024_66086_MOESM1_ESM.docx]

**SUPPLEMENTARY DATA**

**Application of ion-exchange dynamic conditions in the recovery of precious metals from refining waste**

Karolina Goc^1,2,*^, Grzegorz Benke^1^, Joanna Kluczka^2^, Karolina Pianowska^1,2^, Joanna Malarz^1^, Katarzyna Leszczyńska-Sejda^1^

^1^Hydroelectrometallurgy Centre, Łukasiewicz Research Network-Institute of Non-Ferrous Metals, Sowińskiego 5, 44-100 Gliwice, Poland

^2^Department of Inorganic Chemistry, Analytical Chemistry and Electrochemistry, Faculty of Chemistry, Silesian University of Technology, B. Krzywoustego 6, 44-100 Gliwice, Poland

*Corresponding author, tel.: +48 32 238 06 76,
e-mail address: karolina.goc@imn.lukasiewicz.gov.pl

**Table SD-1.** Results of the continuous sorption test for Puromet MTS9200.

| **Puromet MTS9200** | | | | | |
| --- | --- | --- | --- | --- | --- |
| **BV** | **V_final_ [cm^3^]** | **Concentration after sorption [mg/dm^3^]** | | | |
|  |  | **Pt** | **Pd** | **Rh** | **Au** |
| **(1)** | **(2)** | **(3)** | **(4)** | **(5)** | **(6)** |
| 0 | 0 | 0.0 | 0.0 | 0.0 | 0.0 |
| 20 | 100 | 0.2 | 0.1 | 6.3 | 0.1 |
| 40 | 97 | 0.3 | 0.1 | 7.9 | 0.1 |
| 60 | 98 | 0.3 | 0.1 | 9.4 | 0.1 |
| 80 | 103 | 1.0 | 0.4 | 10.3 | 0.1 |
| 100 | 100 | 0.2 | 0.1 | 6.3 | 0.1 |
| 120 | 109 | 0.3 | 0.1 | 7.7 | 0.1 |
| 140 | 106 | 0.3 | 0.1 | 8.6 | 0.1 |
| 160 | 101 | 0.4 | 0.1 | 7.6 | 0.1 |
| 180 | 104 | 0.4 | 0.1 | 6.8 | 0.1 |
| 200 | 100 | 0.4 | 0.1 | 7.7 | 0.1 |
| 220 | 102 | 0.7 | 0.1 | 8.4 | 0.1 |
| 240 | 104 | 2.6 | 1.0 | 8.8 | 0.1 |
| 260 | 100 | 2.9 | 0.7 | 8.2 | 0.1 |
| 280 | 101 | 3.6 | 0.6 | 9.2 | 0.1 |
| 300 | 102 | 3.1 | 0.4 | 9.5 | 0.1 |
| 320 | 100 | 4.0 | 0.7 | 10.1 | 0.1 |
| 340 | 96 | 1.8 | 0.1 | 8.7 | 0.1 |
| 360 | 102 | 3.8 | 0.2 | 10.1 | 0.1 |
| 380 | 101 | 5.5 | 0.4 | 11.2 | 0.1 |
| 400 | 108 | 11.7 | 2.8 | 12.4 | 0.1 |
| 420 | 116 | 8.4 | 0.6 | 10.5 | 0.1 |
| 440 | 105 | 10.4 | 1.1 | 12 | 0.1 |
| 460 | 101 | 11.7 | 1.6 | 12.3 | 0.1 |
| 480 | 95 | 12.5 | 2.6 | 11.1 | 0.1 |
| 500 | 104 | 15.0 | 0.1 | 16.5 | 0.1 |
| 520 | 109 | 22.7 | 1.1 | 19.8 | 0.1 |
| 540 | 105 | 24.8 | 1.6 | 20.5 | 0.1 |
| 560 | 97 | 21.6 | 2.3 | 17.9 | 0.1 |
| 580 | 100 | 23.3 | 2.6 | 17.8 | 0.1 |
| 600 | 102 | 25.1 | 3.5 | 18.4 | 0.1 |
| 620 | 100 | 30.3 | 5.6 | 20.6 | 0.1 |
| 640 | 104 | 34.8 | 9.5 | 21.3 | 0.1 |
| 660 | 108 | 28.6 | 4.1 | 18.6 | 0.1 |
| 680 | 114 | 34.9 | 8.7 | 20.5 | 0.1 |
| 700 | 94 | 36.2 | 11.0 | 21.1 | 0.1 |
| 720 | 101 | 35.5 | 12.3 | 19.2 | 0.1 |
| 740 | 97 | 32.2 | 4.2 | 19.0 | 0.1 |
| 760 | 118 | 41.8 | 12.9 | 21.9 | 0.1 |
| 780 | 104 | 43.5 | 16.6 | 21.8 | 0.1 |
| 800 | 100 | 40.2 | 18.7 | 19.9 | 0.8 |

| **(1)** | **(2)** | **(3)** | **(4)** | **(5)** | **(6)** |
| --- | --- | --- | --- | --- | --- |
| 820 | 110 | 39.1 | 1.4 | 20.4 | 0.1 |
| 840 | 104 | 44.5 | 5.0 | 22.1 | 0.1 |
| 860 | 105 | 48.3 | 8.3 | 22.4 | 0.1 |
| 880 | 105 | 41.8 | 10.2 | 19.5 | 0.1 |
| 900 | 96 | 42.1 | 1.7 | 20.8 | 0.1 |
| 920 | 107 | 51.9 | 8.7 | 23.2 | 0.1 |
| 940 | 107 | 54.7 | 15.3 | 23.6 | 0.1 |
| 960 | 100 | 52.4 | 18.8 | 22.4 | 0.1 |
| 980 | 106 | 46.7 | 2.2 | 19.3 | 0.1 |
| 1000 | 110 | 52.2 | 10.0 | 20.8 | 0.1 |
| 1020 | 116 | 55.7 | 17.3 | 21.2 | 0.1 |
| 1040 | 103 | 39.6 | 15.6 | 15.3 | 0.1 |
| 1060 | 101 | 50.7 | 4.2 | 20.4 | 0.1 |
| 1080 | 103 | 53.6 | 12.3 | 21.6 | 0.1 |
| 1100 | 105 | 58.3 | 18.8 | 21.7 | 0.1 |
| 1120 | 116 | 58.8 | 28.2 | 21.2 | 0.1 |
| 1140 | 102.5 | 49.7 | 3.6 | 20.6 | 0.1 |
| 1160 | 96 | 56.3 | 10.3 | 20.6 | 0.1 |
| 1180 | 99 | 55.4 | 7.7 | 22.1 | 0.1 |
| 1200 | 105 | 59.1 | 15.5 | 22.1 | 0.1 |
| 1220 | 97 | 48.7 | 3.8 | 19.4 | 0.1 |
| 1240 | 97 | 58.2 | 11.3 | 19.4 | 0.1 |
| 1260 | 106 | 60.5 | 19.5 | 23.1 | 0.1 |
| 1280 | 103 | 61.5 | 25.0 | 23.1 | 0.1 |
| 1300 | 102.5 | 53.7 | 4.4 | 19.2 | 0.1 |
| 1320 | 96.5 | 57.2 | 13.5 | 19.2 | 0.1 |
| 1340 | 96 | 58.8 | 20.0 | 19.2 | 0.1 |
| 1360 | 106 | 59.7 | 28.8 | 19.2 | 0.1 |
| 1380 | 101 | 55.3 | 12.2 | 19.7 | 0.1 |
| 1400 | 98 | 58.3 | 24.4 | 19.7 | 0.1 |
| 1420 | 96 | 60.3 | 31.3 | 19.7 | 0.1 |
| 1440 | 107 | 60.1 | 34.1 | 19.7 | 0.1 |

**Table SD-2.** Results of the continuous sorption test for Puromet MTS9850.

| **Puromet MTS9850** | | | | | |
| --- | --- | --- | --- | --- | --- |
| **BV** | **V_final_ [cm^3^]** | **Concentration after sorption [mg/dm^3^]** | | | |
|  |  | **Pt** | **Pd** | **Rh** | **Pt** |
| **(1)** | **(2)** | **(3)** | **(4)** | **(5)** | **(6)** |
| 0 | 0 | 0.0 | 0.0 | 0.0 | 0.0 |
| 20 | 100 | 0.4 | 0.1 | 5.1 | 0.1 |
| 40 | 99 | 0.5 | 0.1 | 7.7 | 0.1 |
| 60 | 101 | 0.6 | 0.1 | 9.3 | 0.1 |
| 80 | 108 | 0.6 | 0.1 | 9.4 | 0.1 |
| 100 | 105 | 0.6 | 0.1 | 5.9 | 0.1 |
| 120 | 113 | 0.6 | 0.1 | 7.5 | 0.1 |
| 140 | 104 | 0.6 | 0.1 | 8.2 | 0.1 |
| 160 | 101 | 0.6 | 0.1 | 7.5 | 0.1 |
| 180 | 109 | 0.7 | 0.1 | 6.0 | 0.1 |
| 200 | 99 | 0.7 | 0.1 | 7.0 | 0.1 |
| 220 | 99 | 0.7 | 0.1 | 7.6 | 0.1 |
| 240 | 103 | 0.7 | 0.1 | 7.5 | 0.1 |
| 260 | 114 | 0.6 | 0.1 | 5.8 | 0.1 |
| 280 | 103 | 0.6 | 0.1 | 7.0 | 0.1 |
| 300 | 99 | 0.6 | 0.1 | 7.5 | 0.1 |
| 320 | 104 | 0.6 | 0.1 | 6.9 | 0.1 |
| 340 | 101 | 0.6 | 0.1 | 6.2 | 0.1 |
| 360 | 106 | 0.7 | 0.1 | 7.7 | 0.1 |
| 380 | 103 | 0.8 | 0.1 | 8.2 | 0.1 |
| 400 | 108 | 0.8 | 0.1 | 7.5 | 0.1 |
| 420 | 106 | 0.8 | 0.1 | 6.4 | 0.1 |
| 440 | 109 | 0.8 | 0.1 | 7.8 | 0.1 |
| 460 | 107 | 0.8 | 0.1 | 8.1 | 0.1 |
| 480 | 96 | 0.8 | 0.1 | 7.5 | 0.1 |
| 500 | 112 | 0.8 | 0.1 | 9.0 | 0.1 |
| 520 | 119 | 1.0 | 0.1 | 10.8 | 0.1 |
| 540 | 114 | 1.0 | 0.1 | 11.8 | 0.1 |
| 560 | 109 | 0.7 | 0.2 | 7.8 | 0.1 |
| 580 | 107 | 0.9 | 0.1 | 9.5 | 1.1 |
| 600 | 108 | 1.2 | 0.1 | 10.9 | 0.1 |
| 620 | 111 | 1.3 | 0.1 | 11.9 | 0.1 |
| 640 | 110 | 1.1 | 0.7 | 10.4 | 0.1 |
| 660 | 120 | 1.1 | 0.1 | 10.5 | 0.1 |
| 680 | 110 | 1.5 | 0.2 | 11.9 | 0.1 |
| 700 | 96 | 1.5 | 0.3 | 12.7 | 0.1 |
| 720 | 107 | 1.1 | 0.7 | 10.2 | 0.1 |
| 740 | 107 | 1.5 | 0.1 | 11.7 | 0.1 |
| 760 | 101 | 1.9 | 0.3 | 13.3 | 0.1 |
| 780 | 99 | 1.9 | 0.3 | 14.4 | 0.1 |
| 800 | 105 | 1.7 | 0.4 | 13.6 | 0.1 |

| **(1)** | **(2)** | **(3)** | **(4)** | **(5)** | **(6)** |
| --- | --- | --- | --- | --- | --- |
| 820 | 101 | 1.6 | 0.1 | 9.7 | 0.1 |
| 840 | 106 | 1.7 | 0.2 | 11.7 | 0.1 |
| 860 | 100 | 1.8 | 0.2 | 13.2 | 0.1 |
| 880 | 105 | 1.7 | 0.3 | 13.2 | 0.1 |
| 900 | 99 | 1.7 | 0.6 | 11.8 | 0.1 |
| 920 | 105 | 2.0 | 0.2 | 13.9 | 0.1 |
| 940 | 97 | 2.5 | 1.2 | 16.5 | 0.1 |
| 960 | 116 | 2.2 | 0.5 | 15.7 | 0.1 |
| 980 | 100 | 2.0 | 0.7 | 12.7 | 0.1 |
| 1000 | 108 | 2.5 | 0.9 | 16.9 | 0.1 |
| 1020 | 120 | 4.6 | 1.3 | 19.9 | 0.1 |
| 1040 | 104 | 3.4 | 1.2 | 15.1 | 0.1 |
| 1060 | 117 | 3.9 | 1.1 | 18.6 | 0.1 |
| 1080 | 116 | 8.3 | 4.0 | 22.2 | 0.1 |
| 1100 | 114 | 13.6 | 8.0 | 23.9 | 0.1 |
| 1120 | 104 | 9.7 | 6.8 | 15.5 | 0.1 |
| 1140 | 98 | 6.4 | 1.8 | 20.9 | 0.1 |
| 1160 | 94 | 13.5 | 5.9 | 20.9 | 0.1 |
| 1180 | 100 | 21.4 | 13.8 | 25.6 | 0.1 |
| 1200 | 101 | 31.5 | 24.3 | 25.6 | 0.1 |
| 1220 | 95 | 26.6 | 13.4 | 25.0 | 0.1 |
| 1240 | 104 | 46.3 | 30.5 | 25.0 | 0.1 |
| 1260 | 99 | 50.2 | 39.5 | 25.2 | 0.1 |
| 1280 | 104 | 55.7 | 46.4 | 25.2 | 0.1 |
| 1300 | 110 | 47.9 | 26.6 | 25.0 | 0.1 |
| 1320 | 99 | 56.7 | 39.1 | 25.0 | 0.1 |
| 1340 | 112 | 59.6 | 45.5 | 25.0 | 0.1 |
| 1360 | 113 | 42.4 | 34.6 | 25.0 | 0.1 |
| 1380 | 107 | 58.9 | 36.0 | 19.9 | 0.1 |
| 1400 | 104 | 61.0 | 45.0 | 19.9 | 0.1 |
| 1420 | 94 | 61.0 | 48.2 | 19.9 | 0.1 |
| 1440 | 94 | 62.5 | 51.0 | 19.9 | 0.1 |

**Table SD-3.** Results of the continuous sorption test for Lewatit MonoPlus MP600.

| **Lewatit MonoPlus MP600** | | | | | |
| --- | --- | --- | --- | --- | --- |
| **BV** | **V_final_ [cm^3^]** | **Concentration after sorption [mg/dm^3^]** | | | |
|  |  | **Pt** | **Pd** | **Rh** | **Pt** |
| **(2)** | **(3)** | **(4)** | **(5)** | **(6)** | **(7)** |
| 0 | 0 | 0.0 | 0.0 | 0.0 | 0.0 |
| 20 | 98 | 0.4 | 0.1 | 5.0 | 0.1 |
| 40 | 99 | 0.5 | 0.1 | 7.5 | 0.1 |
| 60 | 102 | 0.9 | 0.1 | 10.7 | 0.1 |
| 80 | 105 | 1.0 | 0.1 | 11.3 | 0.1 |
| 100 | 105 | 1.2 | 0.4 | 8.4 | 0.1 |
| 120 | 112 | 0.9 | 0.3 | 9.2 | 0.1 |
| 140 | 101 | 0.9 | 0.3 | 10.1 | 0.1 |
| 160 | 94 | 0.9 | 0.3 | 11 | 0.1 |
| 180 | 97 | 1.2 | 0.7 | 9.1 | 0.1 |
| 200 | 98 | 1.0 | 0.6 | 9.0 | 0.1 |
| 220 | 92 | 0.9 | 0.5 | 9.9 | 0.1 |
| 240 | 110 | 1.2 | 0.8 | 11.0 | 0.1 |
| 260 | 97 | 1.1 | 0.5 | 8.8 | 0.1 |
| 280 | 104 | 1.1 | 0.8 | 10.1 | 0.1 |
| 300 | 97 | 1.1 | 0.8 | 11.3 | 0.1 |
| 320 | 105 | 1.0 | 0.6 | 12.0 | 0.1 |
| 340 | 47 | 1.1 | 0.7 | 11.7 | 0.1 |
| 360 | 40 | 0.9 | 0.7 | 11.4 | 0.1 |
| 380 | 103 | 1.4 | 1.0 | 11.7 | 0.1 |
| 400 | 102 | 0.9 | 0.7 | 11.5 | 0.1 |
| 420 | 105 | 2.0 | 1.7 | 13.1 | 0.1 |
| 440 | 104 | 1.2 | 1.0 | 13.6 | 0.1 |
| 460 | 106 | 2.1 | 2.5 | 13.8 | 0.1 |
| 480 | 110 | 1.7 | 2.1 | 14.5 | 0.1 |
| 500 | 107 | 2.0 | 2.3 | 16.8 | 0.1 |
| 520 | 102 | 2.2 | 2.4 | 15.7 | 0.1 |
| 540 | 106 | 4.1 | 5.8 | 21.3 | 0.1 |
| 560 | 104 | 3.8 | 8.4 | 26.7 | 0.1 |
| 580 | 108 | 4.6 | 15.2 | 29.7 | 0.1 |
| 600 | 107 | 5.4 | 26.7 | 27.9 | 0.1 |
| 620 | 102 | 9.9 | 41.6 | 28.0 | 0.1 |
| 640 | 104 | 9.7 | 54.2 | 28.4 | 0.1 |
| 660 | 106 | 10.2 | 60.0 | 27.9 | 0.1 |
| 680 | 104 | 11.9 | 77.7 | 27.3 | 0.3 |
| 700 | 115 | 15.4 | 66.9 | 27.8 | 0.1 |
| 720 | 113 | 14.4 | 80.2 | 26.3 | 0.1 |
| 740 | 96 | 14.2 | 85.3 | 26.1 | 0.1 |
| 760 | 108 | 11.9 | 72.8 | 21.3 | 0.1 |
| 780 | 104 | 23.6 | 72.6 | 25.0 | 0.1 |
| 800 | 100 | 20.2 | 81.4 | 25.0 | 0.1 |

| **(2)** | **(3)** | **(4)** | **(5)** | **(6)** | **(7)** |
| --- | --- | --- | --- | --- | --- |
| 820 | 105 | 19.2 | 85.9 | 24.7 | 0.1 |
| 840 | 106 | 19.2 | 85.9 | 24.7 | 0.1 |
| 860 | 109 | 37.9 | 54.3 | 22.2 | 0.1 |
| 880 | 123 | 26.9 | 70.1 | 24.2 | 0.1 |
| 900 | 99 | 23.5 | 79.7 | 24.0 | 0.1 |
| 920 | 112 | 16.2 | 64.5 | 18.0 | 0.1 |
| 940 | 109 | 34.8 | 68.6 | 22.7 | 0.1 |
| 960 | 103 | 26.5 | 76.5 | 23.3 | 0.1 |
| 980 | 109 | 26.1 | 86.3 | 24.5 | 0.1 |
| 1000 | 103 | 20.2 | 74.1 | 20.8 | 0.1 |
| 1020 | 111 | 30.6 | 63.3 | 19.9 | 0.1 |
| 1040 | 112 | 27.7 | 75.2 | 21.5 | 0.1 |
| 1060 | 116 | 26.3 | 82.3 | 21.7 | 0.1 |
| 1080 | 105 | 18.6 | 61.7 | 16.1 | 0.1 |
| 1100 | 103 | 37.2 | 65.7 | 20.0 | 0.1 |
| 1120 | 103 | 33.1 | 76.3 | 21.7 | 0.1 |
| 1140 | 103 | 31.9 | 82.6 | 22.1 | 0.1 |
| 1160 | 106 | 28.8 | 80.1 | 21.1 | 0.1 |
| 1180 | 100 | 31.4 | 56.5 | 21.2 | 0.1 |
| 1200 | 94 | 30.3 | 68.5 | 21.2 | 0.1 |
| 1220 | 100 | 31.3 | 79.4 | 22.8 | 0.1 |
| 1240 | 103 | 30.5 | 79.5 | 22.8 | 0.1 |
| 1260 | 107 | 44.9 | 58.2 | 20.9 | 0.1 |
| 1280 | 110 | 39.4 | 71.9 | 20.9 | 0.1 |
| 1300 | 111 | 36.6 | 76.5 | 22.5 | 0.1 |
| 1320 | 99 | 32.2 | 71.4 | 22.5 | 0.1 |
| 1340 | 98 | 47.6 | 61.5 | 18.9 | 0.1 |
| 1360 | 104 | 43.3 | 61.5 | 18.9 | 0.1 |
| 1380 | 120 | 39.9 | 61.5 | 18.9 | 0.1 |
| 1400 | 131 | 31.5 | 61.5 | 18.9 | 0.1 |
| 1420 | 106 | 44.5 | 65.2 | 19.6 | 0.1 |
| 1440 | 98 | 43.5 | 65.2 | 19.6 | 0.1 |
| 1460 | 96.5 | 41.9 | 65.2 | 19.6 | 0.1 |
| 1480 | 104 | 41.7 | 65.2 | 19.6 | 0.1 |
